# A Selective Fluorescence Turn-On Probe for the Detection of DCNP (Nerve Agent Tabun Simulant)

**DOI:** 10.3390/ma12182943

**Published:** 2019-09-11

**Authors:** Yuna Jung, Dokyoung Kim

**Affiliations:** 1 Department of Biomedical Science, Graduate School, Kyung Hee University, Seoul 02447, Korea; jungpeng159@gmail.com; 2 Department of Anatomy and Neurobiology, College of Medicine, Kyung Hee University, Seoul 02447, Korea; 3 Center for Converging Humanities, Kyung Hee University, Seoul 02447, Korea; 4 Medical Research Center for Bioreaction to Reactive Oxygen Species and Biomedical Science Institute, School of Medicine, Graduate School, Kyung Hee University, Seoul 02447, Korea

**Keywords:** molecular probe, reaction-based probe, diethylcyanophosphonate, nerve agent, real-time detection

## Abstract

Diethylcyanophosphonate (DCNP) is a simulant of Tabun (GA) which is an extremely toxic chemical substance and is used as a chemical warfare (CW) nerve agent. Due to its toxic properties, monitoring methods have been constantly come under the spotlight. What we are proposing within this report is a next-generation fluorescent probe, **DMHN1**, which allows DCNP to become fully traceable in a sensitive, selective, and responsive manner. This is the first fluorescent turn-on probe within the dipolar naphthalene platform induced by ESIPT (excited state intramolecular proton transfer) suppression that allows us to sense DCNP without any disturbance by other similar G-series chemical weapons. The successful demonstrations of practical applications, such as in vitro analysis, soil analysis, and the development of an on-site real-time prototype sensing kit, encourage further applications in a variety of fields.

## 1. Introduction

**Figure 1:**
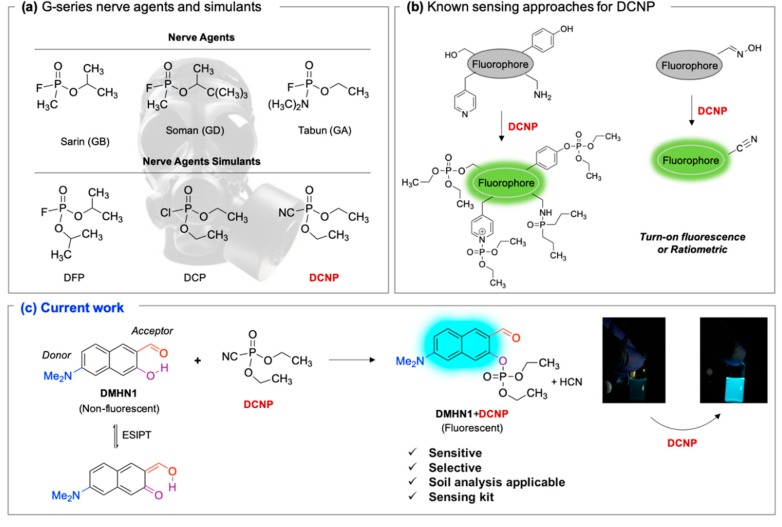
(**a**) G-series nerve agents for chemical warfare and their simulants. (**b**) Representative sensing mechanism of known probes for diethylcyanophosphonate (DCNP). See Table 1 for detail structure and information. (**c**) Sensing mechanism of 6-(dimethylamino)-3-hydroxy-2-naphthaldehyde (**DMHN1)** and DCNP. Schematic illustrations of sensing mode, excited state intramolecular proton transfer (ESIPT) product, merits, and practical applicability. Inset: photos of **DMHN1** in the solution before and after treatment with DCNP under UV light (365 nm).

Nerve agents are a class of organic chemicals that disrupt the central nervous system (CNS) by blocking acetylcholinesterase (AChE), an enzyme that regulates the acetylcholine, a neuro-transmitter [1]. Among them, G-series organophosphonate derivatives [R_1_O(P=O)R_2_OR_3_] such as Sarin (GB), Soman (GD), and Tabun (GA) showed extreme toxic potency, even at very low concentrations (Figure 1a) [2]. G-series nerve agents are historically notorious when used by military regimes and terrorist organizations. Accordingly, analytical tools that can selectively and sensitively detect these kinds of chemical weapons have been developed for the defense, detoxification, and safe management [3,4,5]. Instrument-based methods, such as mass spectrometry, ion-mobility spectroscopy, and photonic crystals, have been used for the sensing, but these methods have several limitations, such as low sensitivity, limited selectivity, time-consuming process, operational complexity, additional sample pre-treatment steps, and non-portability for real-time on-site application [6,7,8,9,10,11]. In this aspect, fluorogenic and chromogenic molecular sensing systems have gained attention due to their advantages for overcoming the limits of current methods [12,13,14,15]. To date, a few fluorescence probes for the detection of diethylcyanophosphonate (DCNP) have been reported, mostly based on chemical reactions (Figure 1b, Figure S1, Table 1): (i) phosphorylation within the moieties such as alkyl-alcohol, phenolic-alcohol, pyridine, and amine and (ii) conversion of hydroxy-imine functional group to nitrile. Some known probes, however, showed drawbacks including low sensitivity and selectivity, complex sensing conditions, and lack of applications (Table 1). In this study, we disclosed a fluorescence turn-on probe based on excited state intramolecular proton transfer (ESIPT)-suppression for the detection of DCNP that showed high sensitivity and selectivity and potential for practical applications. The design of a new sensing platform for the selective detection of DCNP is very challenging due to the similar reactivity of nerve agent simulants.

Recently, we have focused on the development of naphthalene-based electron donor (D)-bridge-acceptor (A)-type dipolar fluorophore and its applications as molecular probes [13,16,17,18]. Within this research process, we found a superior sensing ability of 6-(dimethylamino)-3-hydroxy-2-naphthaldehyde (**DMHN1**) toward DCNP (Figure 1c). The ESIPT causes the fluorescence quenching of **DMHN1** [19] and the nucleophilic substitution reaction between naphthol and DCNP, which may suppress this pathway. Thus, the reaction would be accompanied by emission enhancements as a turn-on type probe. With the discovery of this unique sensing property of **DMHN1** toward DCNP, we systematically analyzed the photophysical properties and demonstrated its applications. Newly developed **DMHN1** showed high sensitivity (8.16 ppm) and selectivity (only DCNP), fast-response time (<3 min), and wide practical applicability, such as in real-time monitoring of DCNP in soil samples.

## 2. Materials and Methods

The chemical reagents were purchased from Aldrich (St. Louis, MO, USA), TCI (Tokyo, Japan), Alfa Aesar (Ward Hill, MA, USA), and Acros Organics (Morris Plains, NJ, USA). Species used to perform the screening of nerve agent simulants: DCNP, DCP, DMMP, TPP, TEP, CH_3_CO_2_H. An aluminum dish (Hanil, Seoul, Korea) and soils (Sand, Clay and Field; Science Love, Goyang, Korea) were purchased for applications. Commercially accessible reagents and anhydrous solvents were used without further purification. Chemical reactions were carried out under argon atmosphere. Thin-layer chromatography (TLC) was performed using pre-coated silica gel 60F-254 glass plates (Merck KGaA, Darmstadt, Germany).

### 2.1. Synthesis

**Figure 2:**
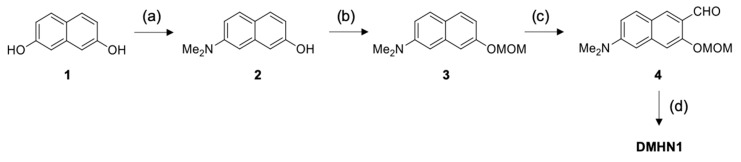
A synthetic scheme for **DMHN1**. (**a**) Na_2_S_2_O_5_, Me_2_NH, DI H_2_O, 150 °C, 3 h, 60%; (**b**) NaH, THF, CH_3_OCH_2_Cl, −15 °C, 7 h, 95%; (**c**) *t*-BuLi, diethyl ether, DMF, −15 °C, 2 h, 52 %. (**d**) *i*PrOH, HCl, 25 °C, 3 h, 90%.

**DMHN1** was synthesized by following the reported method by our group (see Figure 2) [20]. The directed lithiation and formylation are key steps in this synthesis. ^1^H NMR data of synthesized **DMHN1** was compared with the reported result. Important points: (i) Slowly and dropwise add *t*-BuLi (1 drop every 5 s). Fast *t*-BuLi adding generates the isomer and dimer of formylated products which are difficult to separate in column chromatography. (ii) Fresh *t*-BuLi and DMF should be used. (iii) Add *t*-BuLi at a temperature of –20 °C. **Caution**: *t*-BuLi is very reactive and fragile. Keep the appropriate PPE (personal protective equipment).

### 2.2. UV/Vis Absorption and Fluorescence Assay

UV/Vis absorption and fluorescence spectra were acquired by a spectrophotometer (Agilent Technologies Cary 8454, Santa Clara, CA, USA) and spectro-fluorophotometer (SHIMADZU CORP. RF-6000, Kyoto, Japan) with a 1 cm standard quartz cell (internal volume of 1 mL, 108-000-10-40 (10 mm), 108-F-10-40 (10 × 4 mm); Hellma Analytics, Müllheim, Germany) each. The absorption and fluorescence spectra were recorded at the following condition; 10 μM of **DMHN1** in acetonitrile (CH_3_CN, 1% Et_3_N) at 25 °C. Solvent screening was conducted within acetonitrile (ACN), ethanol, isopropanol, dimethylformamide (iPA), dimethyl sulfoxide (DMSO), *N*,*N*-dimethylformamide (DMF), ethyl acetate (EtOAc), deionized water (DI H_2_O), and dichloromethane (DCM, Figures S2 and S3). Photostability of **DMHN1** was monitored under continuous UV light exposure (365 nm, 3 W, Model RM104, Rayman, Goyang, Korea) in CH_3_CN (1% Et_3_N) for 60 min at 25 °C. During the light exposure, UV/Vis absorption and fluorescence change spectra were recorded according to the given time lapse (10 min interval). The maximum absorption wavelength was used for the emission spectra acquirement. High-resolution mass spectra were obtained by a JEOL JMS-700 spectrometer (JEOL, Tokyo, Japan) at the Korea Basic Science Center, Kyung-Pook National University, and the values are reported in units of mass to charge (*m/z*).

### 2.3. Sensing Application for DCNP-Moistened Soils

A spoon of each soil (1 g, sand soil, clay soil, and field, respectively; Science Love, Republic of Korea) was transferred to an aluminum dish (Hanil, 52807, China). Two milliliters of DCNP solution (100 mM) in acetonitrile was poured into an aluminum dish under room temperature (25 °C). Soils were incubated for 2 min at 25 °C. After incubation, each soil sample was transferred into 3 mL of **DMHN1** solution (10 μM) in CH_3_CN (1% Et_3_N). The fluorescence changes of the solution were investigated for 0–120 min by a digital camera (Sony, Alpha A5100, Tokyo, Japan) under UV light (365 nm). The relative fluorescence intensity and standard deviation were calculated by Image-J software (NIH, Bethesda, Rockville, MD, USA) in the specific fluorescence signal region.

### 2.4. Sensing Kit Application

**DMHN1** solution (10 μM, CH_3_CN (1% Et_3_N), 1 mL) was placed in a screw-cap HPLC vial (2 mL size, YL Science, YL-VO1236, Guri, Korea) as a prototype sensing kit. A drop of the original DCNP solution was collected and transferred to the vial by using a capillary tube (Marienfeld, Non-heparinized, Lauda-Königshofen, Germany). The fluorescence changes of the kit were monitored for ~40 s with a digital camera (Sony, Alpha A5100, Tokyo, Japan) under UV light (365 nm). The relative intensity and standard deviation were calculated by Image-J software (NIH, Bethesda, Rockville, MD, USA) in the fluorescence signal region from video.

## 3. Results and Discussion

### 3.1. Sensing Ability of DMHN1 for DCNP

**Figure 3:**
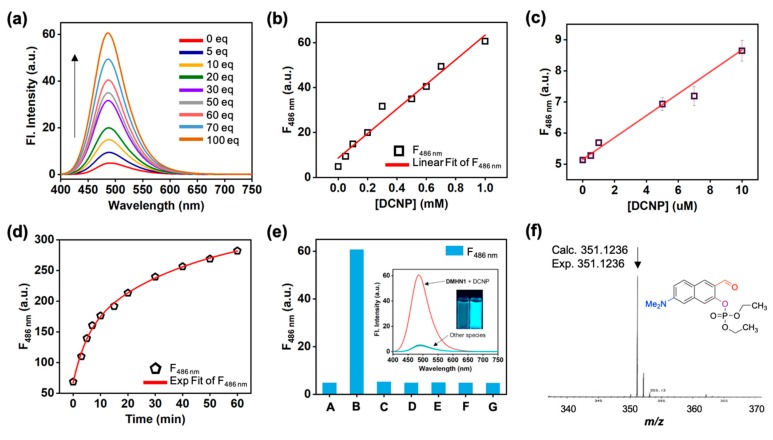
(**a**) Fluorescence change spectra of **DMHN1** (10 μM) measured immediately after adding DCNP (0–100 eq; 0–1 mM) gradually in CH_3_CN (1% Et_3_N) at 25 °C. (**b**) A plot of fluorescence intensity (peak height at 486 nm) changes of **DMHN1** (10 μM) with various concentrations of DCNP (0–1 mM). (**c**) Fluorescence intensity plot of **DMHN1** (10 μM) with a low concentration of DCNP (0.5–10 μM). The emission spectra in the graph (**a**–**c**) were measured after 1 min under excitation at the maximum absorption wavelength. (**d**) A plot of fluorescence intensity (peak height at 486 nm) of **DMHN1** (10 μM) after adding DCNP (1 mM) in CH_3_CN (1% Et_3_N), measured for 60 min at 25 °C. (**e**) Fluorescence changes (peak height at 486 nm) of **DMHN1** (10 μM) measured immediately after adding various organophosphorus compounds (100 eq) in CH_3_CN (1% Et_3_N) at 25 °C. (A) **DMHN1**, (B) DCNP: diethyl cyanophosphonate, (C) DCP: diethyl chlorophosphate, (D) DMMP: dimethyl methylphosphate, (E) TPP: triphenyl phosphate, (F) TEP: triethylphosphate, (G) CH_3_COOH: acetic acid. (**f**) HR-mass spectra of **DMHN1**+DCNP.

A solution of **DMHN1** in sensing media (acetonitrile, 1% Et_3_N; activator of naphthol moiety) exhibited weak fluorescence due to the ESIPT quenching. However, after being treated with DCNP, it showed significant fluorescence enhancement at an emission maximum of 485 nm (Figure 3a) upon excitation at 388 nm. In the screening of sensing media, acetonitrile gave the best response: (i) negligible fluorescence of **DMHN1** itself by ESIPT effect, (ii) significant fluorescence recovery (>20-fold) after reaction with DCNP (Figures S2, S3 and Table S1). In the pH screening (pH 4, 5, 6, 7, 7.4, 8, 9), **DMHN1** showed sensing ability in basic pHs (pH 8, 9), but the signal was not high enough to detect it, compared with the acetonitrile condition (Figure S4). The reasons of these results seem to be (i) activation of naphthol moiety at basic pHs via deprotonation and (ii) decomposition of reactive DCNP in aqueous media. The computational calculation data clearly show the intramolecular H-bonding between the aldehyde and *ortho*-hydroxyl group in the most stable conformational structure of **DMHN1** (Figure S5). The HOMO-LUMO energy differences, 338.11 nm (*Δ*E = 3.67 eV, condition: vacuum), represent the absorption of **DMHN1** in the short wavelength region, and they are corresponding to the experimental results of UV/Vis absorbance; absorption maximum around 350–400 nm (Table S1, condition: within various solvents).

A good linear relationship between the fluorescence intensity of **DMHN1** and DCNP concentration was observed in both high (0–1 mM, Figure 3b, Figure S6) and low concentration ranges (0–10 μM, Figure 3c), and it displayed a high sensitivity; detection limited to 8.16 ppm based on a S/N (signal-to-noise) criteria ratio of more than 3. This value is comparable with the known organophosphorus probes (Table 1). Within the time-course study, we monitored a significant fluorescence enhancement of **DMHN1** with addition of DCNP within 3 min, and it appeared to show further saturation over 20 min (Figure 3d, Figure S7).

The selectivity of **DMHN1** toward DCNP with structurally similar nerve gases including DCP (diethyl chlorophosphate), DMMP (dimethyl methylphosphonate), TPP (tripropyl phosphate), TEP (triethyl phosphate), and acid (acetic acid in this study) was then evaluated (Figure 3e, Figure S8). The nitrile (-CN) leaving group containing DCNP only induced fluorescence enhancement of **DMHN1**, and most of the other simulants showed no change despite the possibility of a SN_2_ type reaction. This superior selectivity is one of the advantages compare with known DCNP probes. To understand the selectivity and sensing mechanism, we analyzed the product using a high-resolution mass spectrometry (HR-mass) and ^31^P NMR. What we mainly observed was the only phosphorylation production (*m/z* = 351.1236, calc. = 351.1236, Figure 3f) (^31^P-NMR in Figure S9), and this result represents that intramolecular H-bonding containing naphthol moiety in **DMHN1** has limited nucleophilicity to attack the electrophilic phosphorous center, in the case of a more reactive cyanide anion bonded DCNP, in comparison to other simulants.

### 3.2. Sensing Application of DMHN1 for DCNP-Moistened Soils

**Figure 4:**
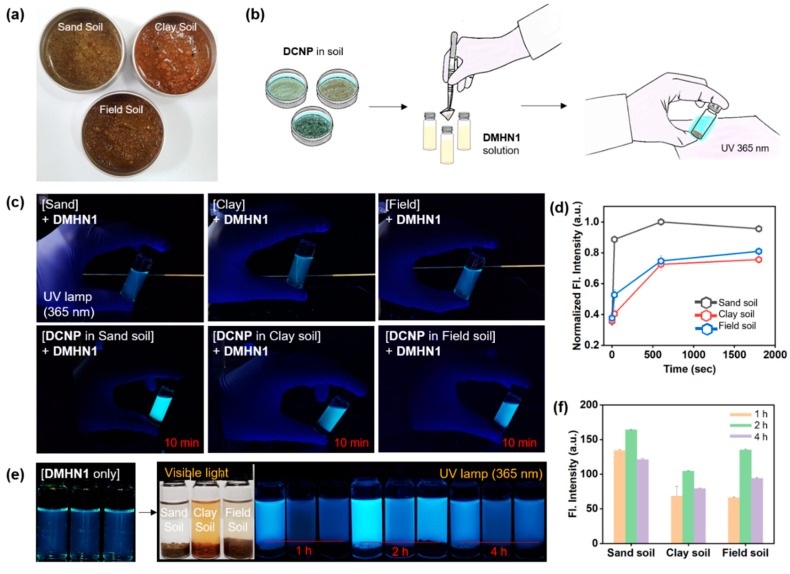
(**a**) Photos of DCNP moistened soils under natural light. (**b**) Schematic illustration of the soil test. DCNP moistened soils transferred to a vial of CH_3_CN (1% Et_3_N) (3 mL) containing **DMHN1** (10 μM). (**c**) Photos of **DMHN1** (top, 10 μM) in CH_3_CN (1% Et_3_N) and after adding DCNP moistened soils (bottom, 1 g, DCNP content: 100 mM). These photos were taken after 10 min at 25 °C. (**d**) The fluorescence emission changes of solutions are shown in panel (**c**) after the soil settled. The relative intensity was calculated by Image-J software. (**e**) Photos of **DMHN1** (10 μM) after adding each soil (1 g) moistened with DCNP (100 mM) in CH_3_CN (1% Et_3_N) under UV light (365 nm). (**f**) Fluorescence intensity plot of solutions as shown in panel (**e**) after the soil settles. The relative intensity was obtained by Image-J software.

Given that **DMHN1** is highly selective and sensitive towards DCNP, we demonstrated the practical applicability of **DMHN1**. For the first demonstration, we used **DMHN1** within various soil samples (sand, clay, and field) for the detection of DCNP, because chemical warfare nerve gas is usually sprayed on the field during wartime or a terrorist attack. The protocol: step 1, put 1 g of each soil (sand, clay, and field, respectively) into an aluminum dish; step 2, treat the DCNP solution (100 mM in CH_3_CN); step 3, pour DCNP-pretreated soils (1 g) into the solution of **DMHN1** (10 μM, 3 mL in CH_3_CN, 1% TEA); and step 4, monitor fluorescence changes at the ambient temperature (25 °C) (Figure 4a,b). Within a few seconds, a significant fluorescence emission was observed in all soil samples under UV light (365 nm) (Figure 4c, Figure S10), and the signal response became saturated within 60 min (Figure 4d–f). The sand soil showed dramatic changes within a few seconds, and the others showed sufficient responses within 10 min. In the concentration-dependent sensing assay of DCNP in the soil samples, **DMHN1** (10 μM) represented the detection limits as 3.125 mM for sand soil and 6.25 mM for the other soils (Figure S11). These differences were derived from the character of soils; surface area, particle size, dispersity, and a light scattering in the given solvent. These practical application results provide that the **DMHN1** can be applied in the detection of DCNP in environmental samples, particularly within soils.

### 3.3. Sensing Kit Test for Real-Time Detection of DCNP

**Figure 5:**
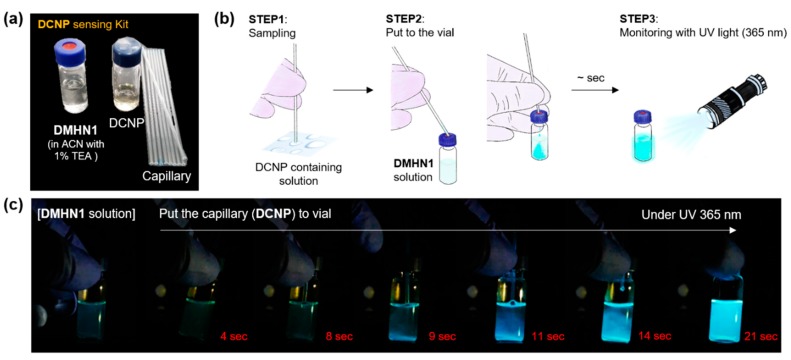
(**a**) Photos of DCNP sensing kit under natural light. (**b**) Schematic illustration of the DCNP sensing kit. (**c**) Photos of **DMHN1** (10 μM) in CH_3_CN (1% Et_3_N) and the progression after adding DCNP. Photos were taken from the video clip (Movie S1) at 2, 9, 11, 14, and 21 s at 25 °C.

To utilize the potential of **DMHN1**, we prepared a prototype DCNP sensing kit for real-time on-site application. Prior to the kit development, a high photostability of **DMHN1** was verified under strong light irradiation (365 nm, 3 W, 1 h) (Figure S12). The components of the DCNP sensing kit were: (i) **DMHN1** solution (10 μM in CH_3_CN, 1% Et_3_N), (ii) DCNP crude solution for positive control, and (iii) capillary (diameter: 1.2 mm) for the liquid sample collection (boiling point of GA: 247.5 °C at 477.5 °F, DCNP: 104 °C/19 mm Hg (lit.)) (Figure 5a). The protocol: step 1, sampling the solution, which contain DCNP, by using a capillary; step 2, put the capillary into the vial through the polytetrafluoroethylene (PTFE)/silicon rubber screw cap and shake (by hand) after removing the capillary; and step 3, monitor the fluorescence changes with a hand-held 365 m UV lamp (Figure 5b). In order to verify these changes, we added the DCNP-positive control solution to the **DMHN1** solution using the capillary (approximately 20 μL) and analyzed the fluorescence responses. Surprisingly, the bright blue fluorescence was monitored from 9 s and it became saturated within 21 s (Figure 5c, Figure S13, and SI Movie 1). With the current state of our prototype sensing kit, we could selectively and sensitively detect DCNP without sample pre-treatment in a real-time on-site situation. The next step of this study is to test the kit in a situation that requires handling the actual nerve agent, GA.

## 4. Conclusions

In conclusion, we developed a selective fluorescence turn-on probe, **DMHN1**, that allows the tracing of DCNP, a simulant of GA nerve gas. As a next-generation fluorescence probe, **DMHN1** showed superior sensing ability of DCNP with high selectivity and sensitivity (8.16 ppm) and fast response time (<3 min), and it can be used in a real-time on-site situation. The sensing application of **DMHN1** in the DCNP-moistened soils and the development of a proto-type sensing kit proved its potential for further studies. Within this study, the first new sensing approach was disclosed; fluorescent turn-on by reaction-based suppression of ESIPT fluorescence quenching in the intramolecular H-bonding containing D-A-type fluorophore. The fast and selective sensing abilities of **DMHN1** encourage further applications in basic science as well as at war zone and crime scenes.

## Figures and Tables

**Table 1:** Summary of known fluorescent probes for DCNP. * n.r.: not reported; DFP: diisopropylfluorophosphate; DCP: diethyl chlorophosphate; DEMP: diethyl methylphosphonate; DMSO-TEA: Dimethyl sulfoxide-triethylamine; HEPES: 4-(2-hydroxyethyl)-1-piperazineethanesulfonic acid; THF: tetrahydrofuran; PBS: phosphate-buffered saline; MES: 2-(N-morpholino)ethanesulfonic acid; DMAP: 4-dimethylaminopyridine.

Probes	λ_ex_/λ_em_ (nm) Sensing Media)	Detection Limit	Selectivity	Reaction Condition (time)	Application	Reference
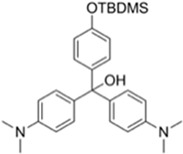	266/n.r. (CH_3_CN-H_2_O)	30 ppm	○ (Multi-sensing: DFP)	25 °C (30 s)	Polyurethane membrane vapor test	[21]
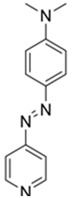	475/n.r. (CH_3_CN-H₂O)	0.9 mM	○ (Multi-sensing: DCP)	25 °C (10 min)	Polyurethane film vapor test	[22]
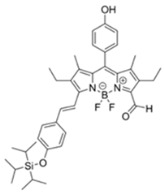	555/625 (CH_3_CN)	0.91 ppm (visible), 0.36 ppm (Fl)	○ (Multi-sensing: DFP)	25 °C (n.r.)	Silica gel plate and polyethylene oxide membrane vapor test	[23]
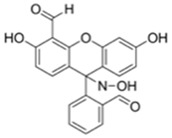	410/n.r. (DMSO-TEA)	3 mM	○ (Multi-sensing: DCP)	25 °C (60 min)	Chemogenic response test	[24]
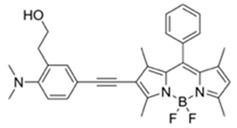	530/555 (CH_3_CN-H₂O)	2.7 ppm (in CH_3_CN)	○ (Multi-sensing: DFP	25 °C (n.r.)	Hydrogel coated polyethylene strip vapor test, silica strip test	[25]
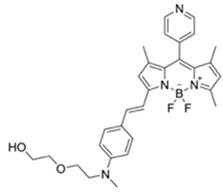	530/575 (CH_3_CN)	4.52 ppm (visible), 4.01 ppm(Fl)	○ (Multi-sensing: DFP)	25 °C (n.r.)	Silica gel plate test, polyethylene oxide membrane vapor test	[26]
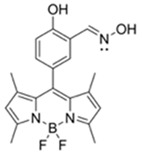	499/508 (HEPES buffer)	92.2 μM	○ (Multi-sensing: DCP, DEMP)	n.r.	n.r.	[27]
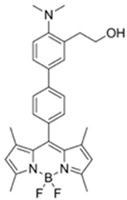	470/507 (CH_3_CN)	7 ppm (CH_3_CN), 4 ppm (H₂O-CH_3_CN)	○	25 °C (~ s)	Hydrogel coated polyethylene strip vapor test	[28]
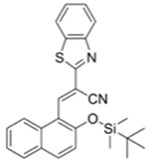	320, 410/486 (CH_3_CN-H₂O)	3.09 μM	○ (Relay sensing with F^-^ ion)	25 °C (~ s)	TLC plate emerging test	[29]
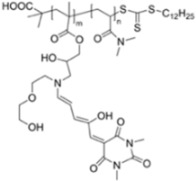	550/n.a. (Dioxane)	1 mM	○	n.r.	Vapor test	[30]
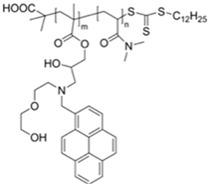	345/375-395 (THF-H₂O)	0.1 mM	○ (Multi-sensing)	n.r.	Quartz plate vapor test	[31]
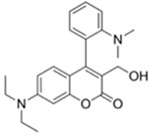	388/460 (CHCl_3_)	0.044 nM (DCP)	× (selective for DCP)	25 °C (5 min)	n.r.	[32]
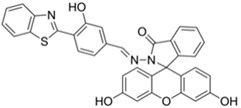	365/430, 559 (DMSO-TEA)	1.6 μM (DCP)	× (selective for DCP)	25 °C (n.r.)	Vapor test	[33]
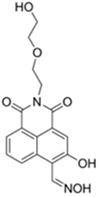	458/570 (PBS buffer)	21.9 nM	○ (Multi-sensing: DCP)	25 °C (10 min)	Silica plate vapor test	[34]
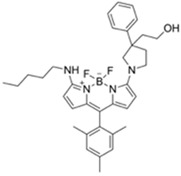	550/635 (MES buffer)	90.8 pM	×	25 °C (10 min)	Water test	[35]
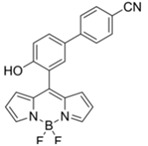	480/520 (DMF-TEA/ DMAP)	20.7 ppb (DCP)	× (selective for DCP)	25 °C (10 min)	Paper strip test	[36]
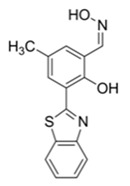	410/480 (DMF)	1.3 nM	○	25 °C (4 min)	Vapor test	[37]
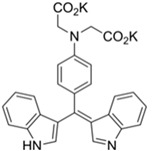	465/550 (H₂O)	10.8 μM	○	25 °C (30 min)	Paper test vapor test	[38]
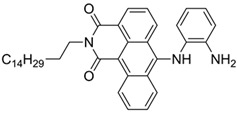	520/588 (CHCl_3_-TEA)	88 nM (DCP) 72 μM (Phosgene)	○	25 °C (2 min)	Polystyrene membrane vapor test	[39]

## Supplementary Materials

The following are available online at https://www.mdpi.com/1996-1944/12/18/2943/s1, Supplementary Figures: representative sensing mechanism of known DCNP probes, synthetic scheme, UV/Vis absorption and fluorescence spectra, quantum chemical calculation, NMR analysis, and HR-mass spectra.

## Abbreviations

DCNP	Diethylcyanophosphonate
DCP	Diethyl chlorophosphate
DMMP	Dimethyl methylphosphonate
TPP	Tripropyl phosphate
TEP	Triethyl phosphate
GA	Tabun
GB	Sarin
GD	Soman
CW	Chemical warfare
ESIPT	Excited state intramolecular proton transfer
CNS	Central nervous system
AChE	Acetylcholinesterase
ACN	Acetonitrile
EtOH	Ethanol
iPA	Isopropanol
DMSO	Dimethyl sulfoxide
DMF	*N*,*N*-dimethylformamide
EtOAc	Ethyl acetate
DI H_2_O	Deionized water
DCM	Dichloromethane
PTFE	Polytetrafluoroethylene
	
