# Supplementary material for: A Selective Fluorescence Turn-On Probe for the Detection of DCNP (Nerve Agent Tabun Simulant)
Source: Materials (Basel). 2019 Sep 11;12(18):2943. doi: 10.3390/ma12182943 (PMC6766206; doi:10.3390/ma12182943)
Supplement: Supplementary file 1 [file materials-12-02943-s001.zip › materials-575528-SI.pdf]

materials

Communication

A Selective Fluorescence Turn-On Probe for the Detection of DCNP (Nerve Agent Tabun Simulant)

Yuna Jung [1] and Dokyoung Kim [1,2,3,4,*]

[1] Department of Biomedical Science, Graduate School, Kyung Hee University, Seoul 02447, Korea; jungpeng159@gmail.com (Y.J.); dkim@khu.ac.kr (D.K.)
[2] Department of Anatomy and Neurobiology, College of Medicine, Kyung Hee University, Seoul 02447, Korea
[3] Center for Converging Humanities, Korea University, Seoul 02841, Korea
[4] Medical Research Center for Bioreaction to Reactive Oxygen Species and Biomedical Science Institute, School of Medicine, Graduate School, Kyung Hee University, Seoul 02841, Korea
* Correspondence: dkim@khu.ac.kr; Tel.: +82-02-961-0297

Received: 01 August 2019; Accepted: 09 September 2019; Published: date

Supporting Figures

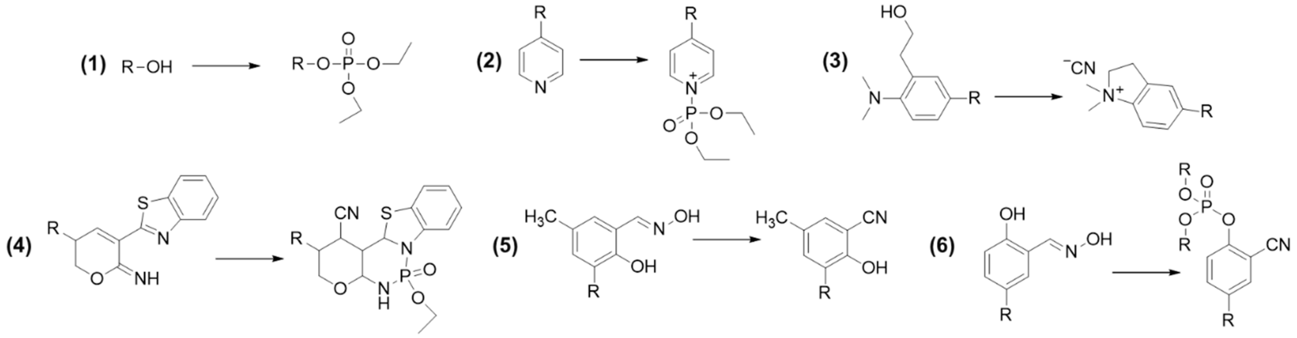

Figure 1. Representative sensing mechanism of known probes for DCNP.

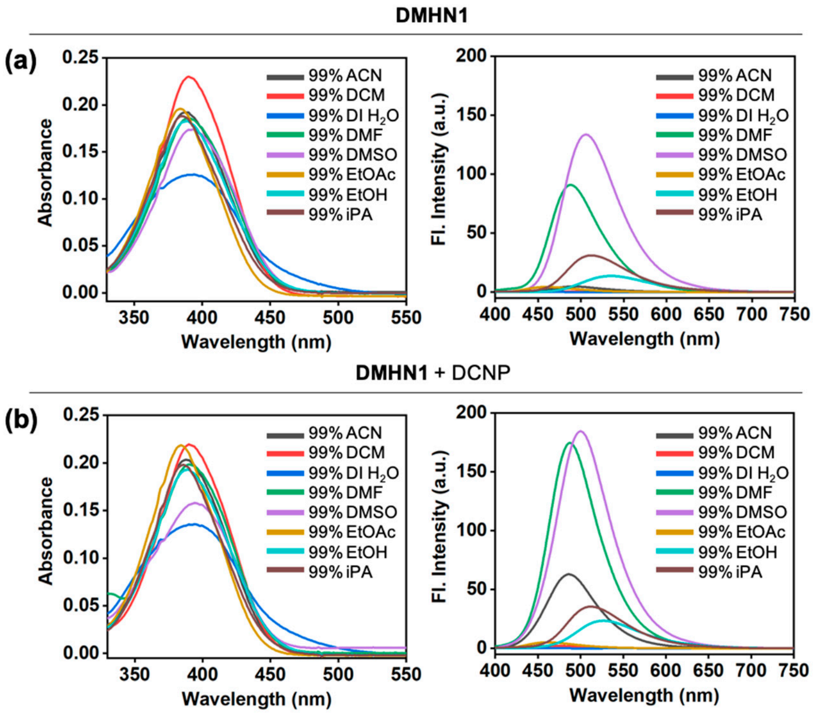

Figure 2. DCNP sensing properties of **DMHN1**. (**a**) UV/vis absorption and emission spectra of **DMHN1** (10 µM) and (**b**) **DMHN1** (10 µM) upon addition of DCNP (right; 1 mM) in various solvents (99% ACN, DCM, DI H_2O, DMF, DMSO, EtOAc, EtOH, and iPA with 1% Et_3N). All the measurements were carried out 25 °C without incubation in the given solvents. The spectra were recorded under excitation at the maximum absorption wavelength.

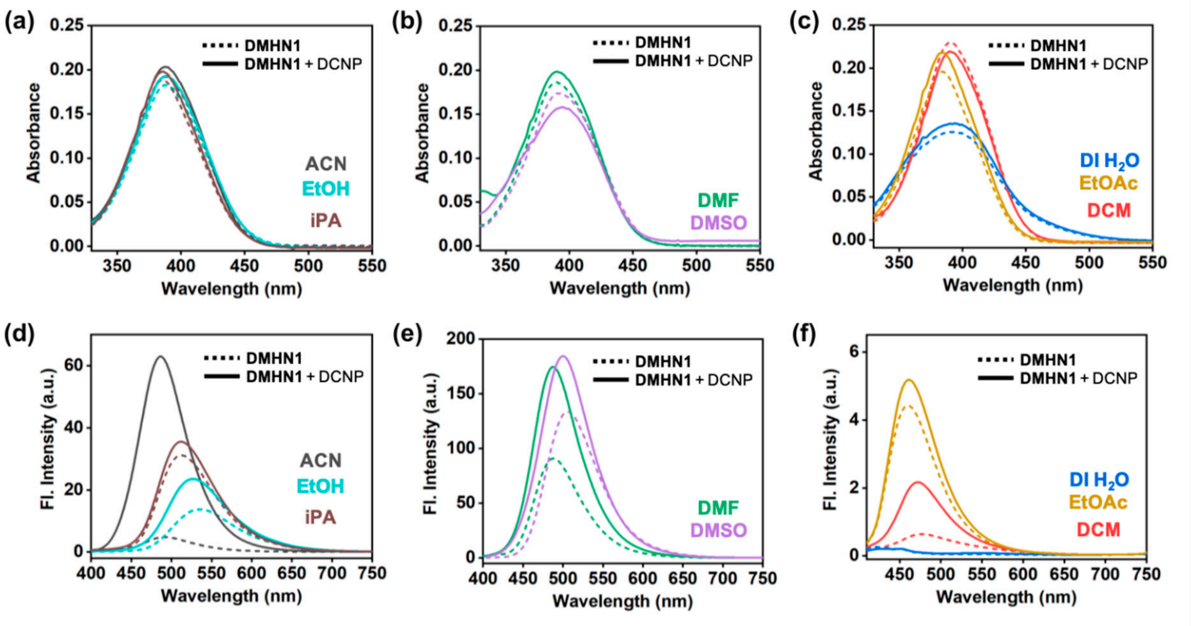

Figure 3. DCNP sensing properties of **DMHN1**. (**a**, **b**, **c**) absorption and (**d**, **e**, **f**) emission spectra of **DMHN1** (10 µM) and **DMHN1** (10 µM) upon addition of DCNP (right; 1 mM) in the given solvents (99% ACN, DCM, DI H_2O, DMF, DMSO, EtOAc, EtOH, and iPA with 1% Et_3N). All the measurements were carried out 25 °C in the given solvents. The spectra were recorded under excitation at the maximum absorption wavelength.

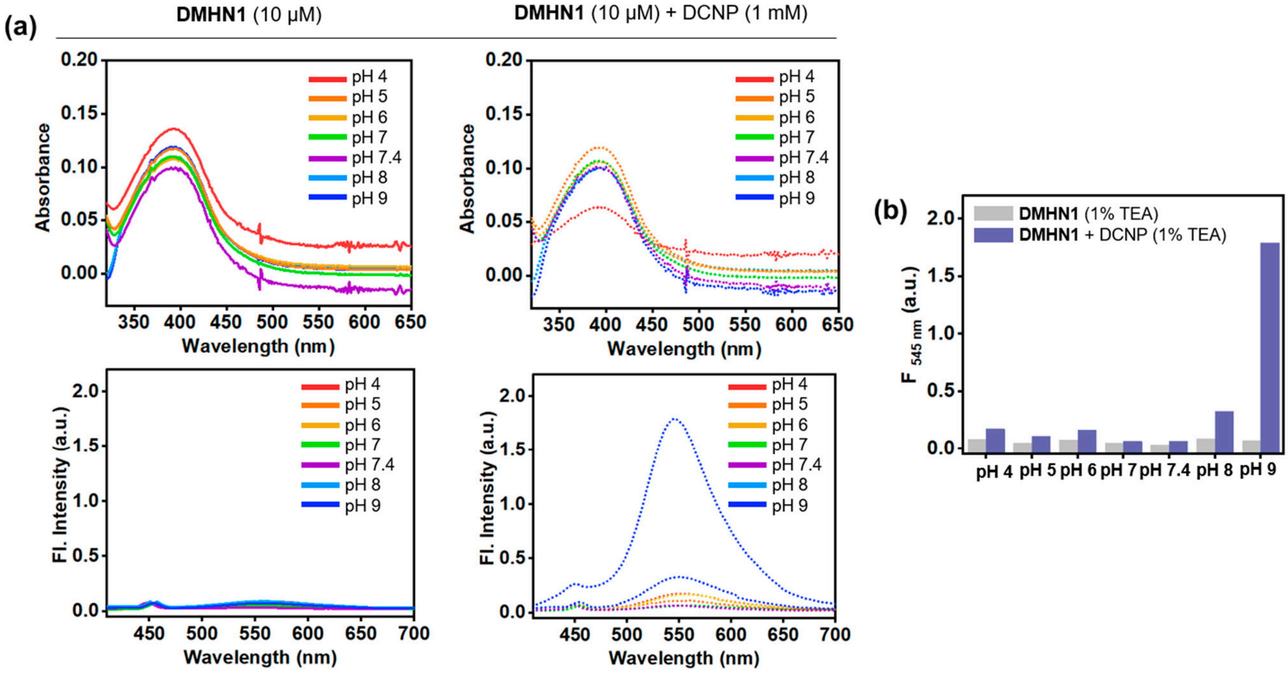

Figure 4. pH dependent screening of **DMHN1**. (**a**) UV/vis absorption and emission spectra of **DMHN1** (10 µM) upon addition of DCNP (1 mM) in various pH buffers (pH 4, 5, 6, 7, 7.4, 8, 9) without incubation at 25 °C. (**b**) Fluorescence intensity changes (peak height at 545 nm) of **DMHN1** (10 µM)

measured directly after adding DCNP (1 mM) in various pH buffers (pH 4, 5, 6, 7, 7.4, 8, 9) without incubation at 25 °C. The emission spectra were measured under excitation at 393 nm.

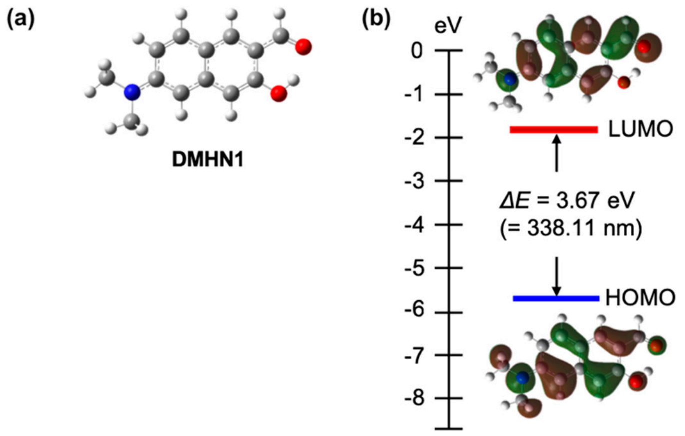

Figure 5. Quantum chemical calculation of **DMHN1**. (**a**) the most stable conformational structure of **DMHN1**. (**b**) The HOMO/LUMO and its energy difference (ΔE, unit: eV) of **DMHN1** obtained by DFT calculations (APFD/6-31+G(d,p)).

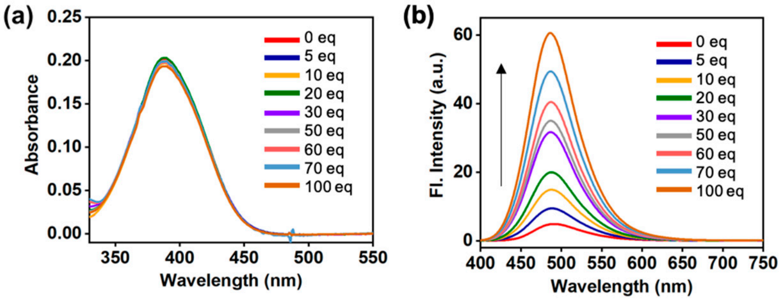

Figure 6. Equivalent-dependent absorption and emission spectra changes of **DMHN1** with DCNP. (**a**) UV/vis absorption and (**b**) emission spectra of **DMHN1** (10 μM) upon gradual addition of DCNP (0–100 eq) in CH_3CN (1% Et_3N), measured immediately at 25 °C. The fluorescence emission spectra were measured under maximum excitation at each equivalent.

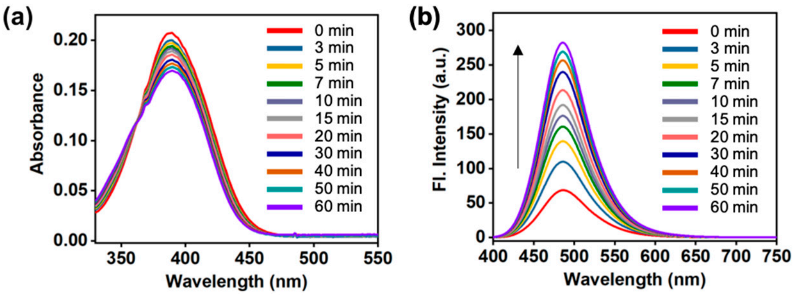

Figure 7. Time-dependent absorption and emission spectra of **DMHN1** with DCNP. (**a**) UV/vis absorption and (**b**) emission spectra changes of **DMHN1** (10 μM) upon addition of DCNP (1 mM) in CH_3CN (1% Et_3N), at 25 °C. The emission spectra were measured under excitation at 390 nm. Each spectrum was recorded at 0–60 min (10 min interval) after mixing with DCNP.

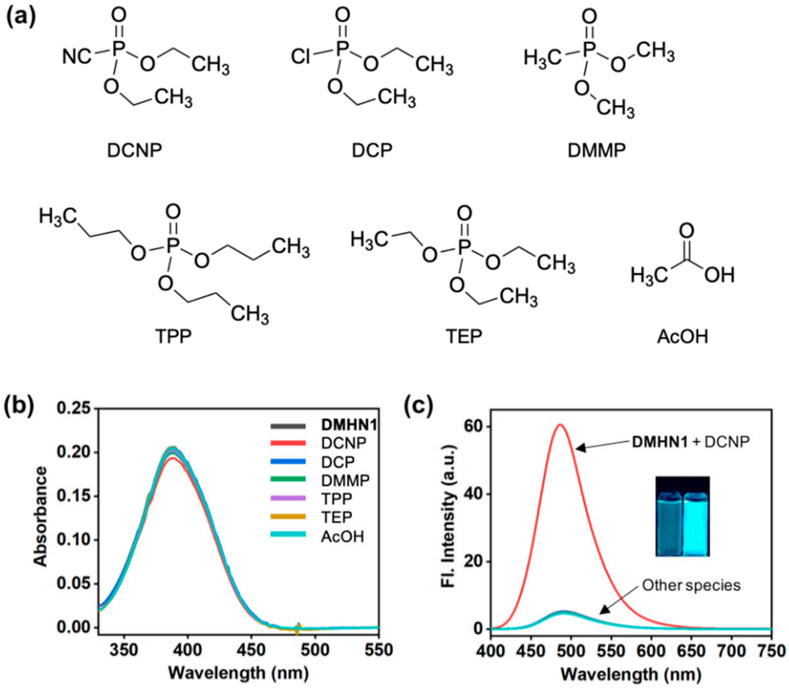

Figure 8. Selectivity assay of **DMHN1**. (**a**) Structure of known chemical warfare nerve agent simulants. (**b**) UV/vis absorption and (**c**) emission spectra of **DMHN1** (10 μM) upon addition of DCNP (1 mM) and each nerve agent simulants (100 eq; 1 mM) in CH_3CN (1% Et_3N), measured without incubation at 25 °C. Inset: Photos of **DMHN1** before and after sensing DCNP under the UV light (365 nm). The emission spectra were measured under excitation at 390 nm.

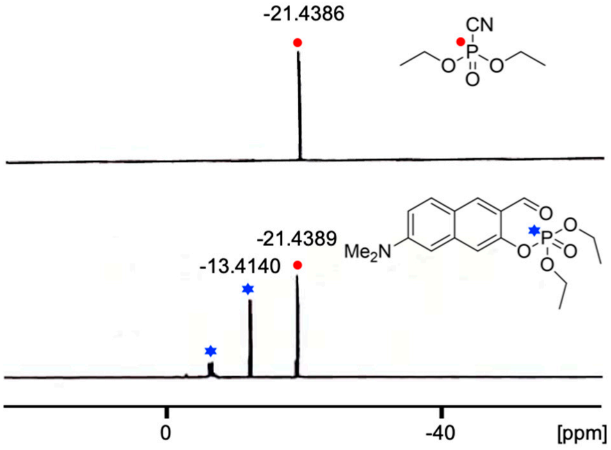

Figure 9. ^{31}P-NMR of DCNP and reaction product (**DMHN1**+DCNP). (Top) DCNP in DMSO-d_6. (Bottom) **DMHN1**+DCNP in DMSO-d_6.

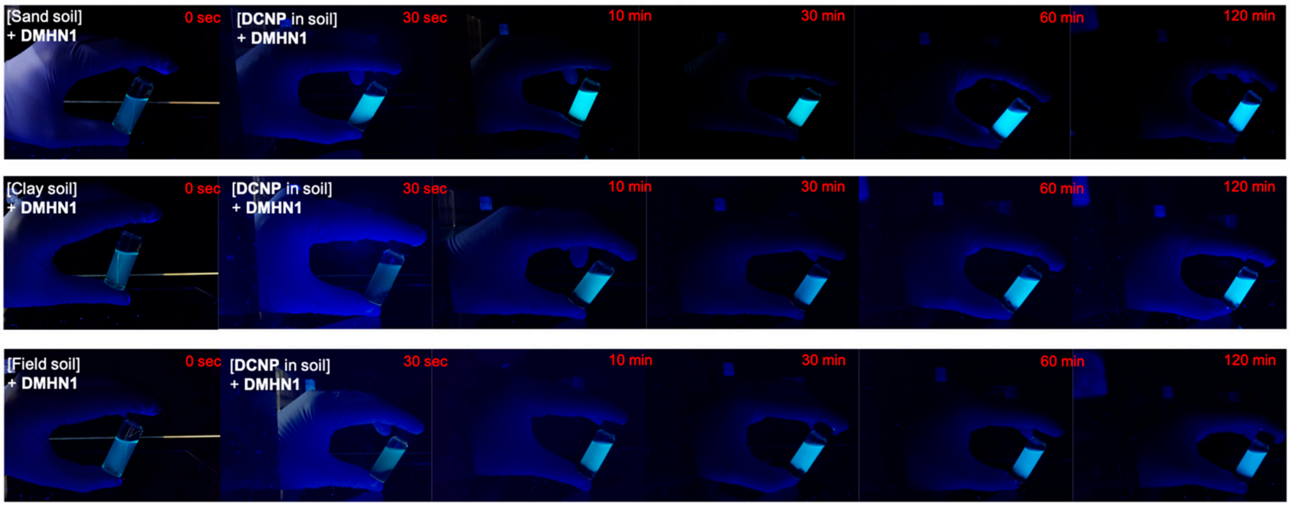

Figure 10. Photographs of **DMHN1** (10 μM) after addition of each soil (1 g of sand, clay, and field) moistened with DCNP (100 mM) in CH_3CN (1% Et_3N) under UV light (365 nm). Photos were taken for 120 min.

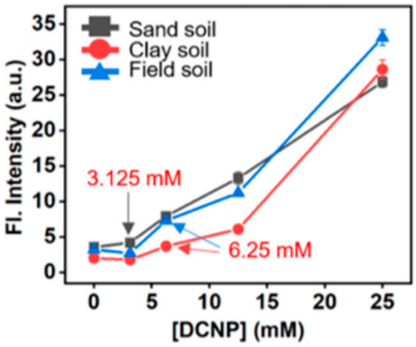

Figure 11. Fluorescence maximum emission plot of **DMHN1** (10 μM) with various concentration of DCNP (0–25 mM) within each soil. The emission spectra were measured after 30 min under excitation at the maximum absorption wavelength.

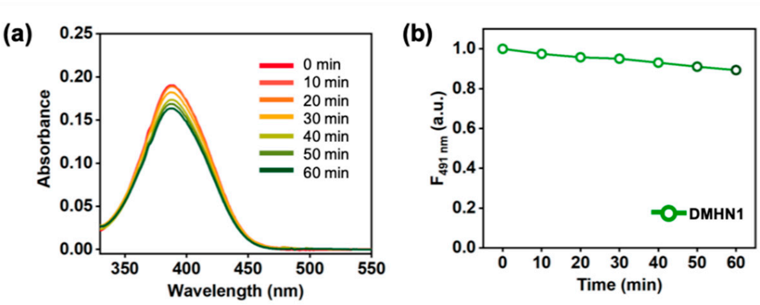

Figure 12. Photostability analysis of **DMHN1**. (**a**) absorption and (**b**) fluorescence intensity of **DMHN1** (10 μM) under the continuous light exposure (UV LED light, 365 nm, 3 W) in CH_3CN (1% Et_3N). The fluorescence intensity was derived from the emission spectra at maximum wavelength (491 nm).

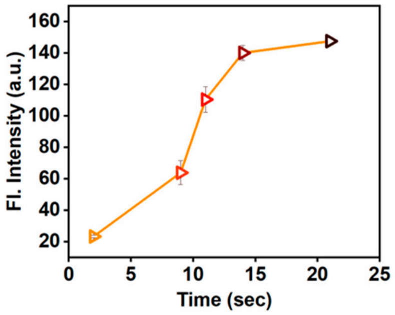

Figure 13. Fluorescence intensity plot of solutions as shown in panel (c) in Figure 5 after expose DCNP solution. The relative intensity was calculated by Image-J software.

Supporting Tables

Table 1. Photophysical property of **DMHN1**. ACN; acetonitrile, DCM; dichloromethane, DI H_2O; deionized water, DMF; *N,N*-dimethylformamide, DMSO; dimethyl sulfoxide, EtOAc; ethyl acetate, EtOH; ethanol, iPA; isopropyl alcohol.

Compounds	Solvents	λ_{abs} (nm)	λ_{fl} (nm)	Stoke's shift
**DMHN1**	ACN	388	491	103
	DCM	390	476	86
	DI H_2O	394	418	24
	DMF	390	488	98
	DMSO	394	506	112
	EtOAc	384	460	76
	EtOH	389	536	147
	iPA	385	512	127

1. H NMR and ^{31}P NMR of DMHN1

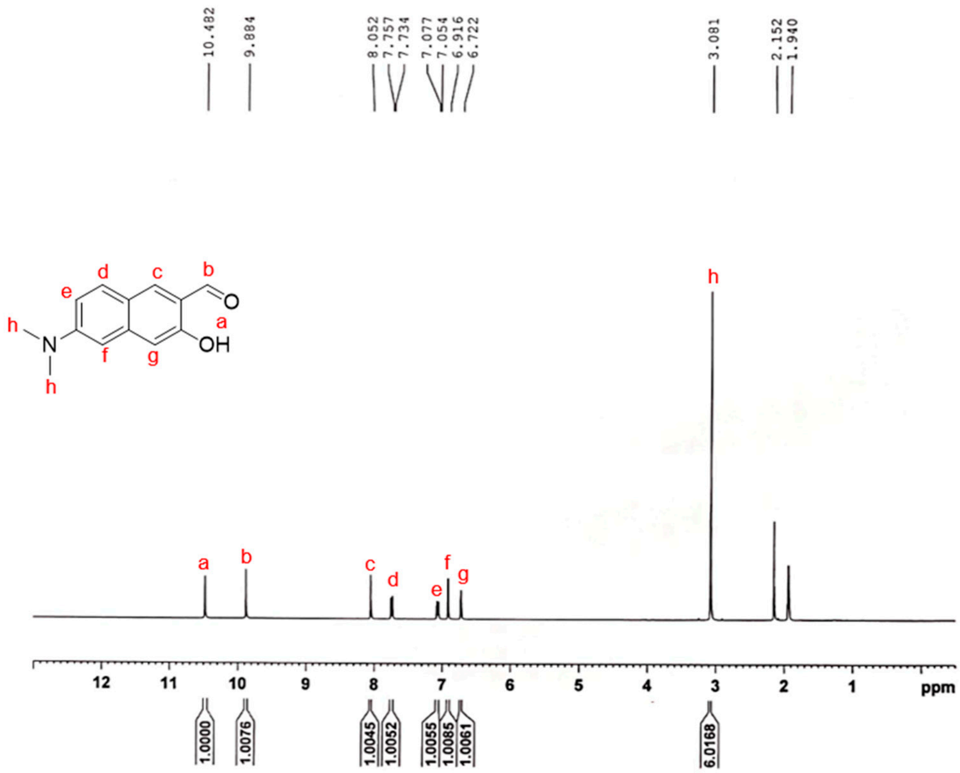

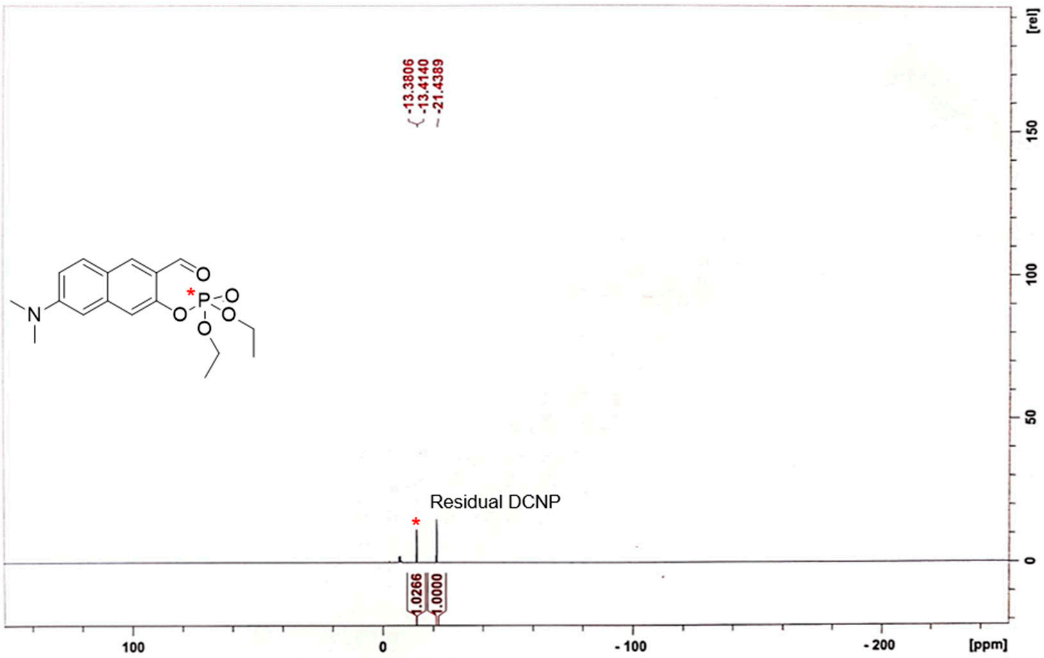

High-resolution mass spectra for DMHN1+DCNP (reactant)

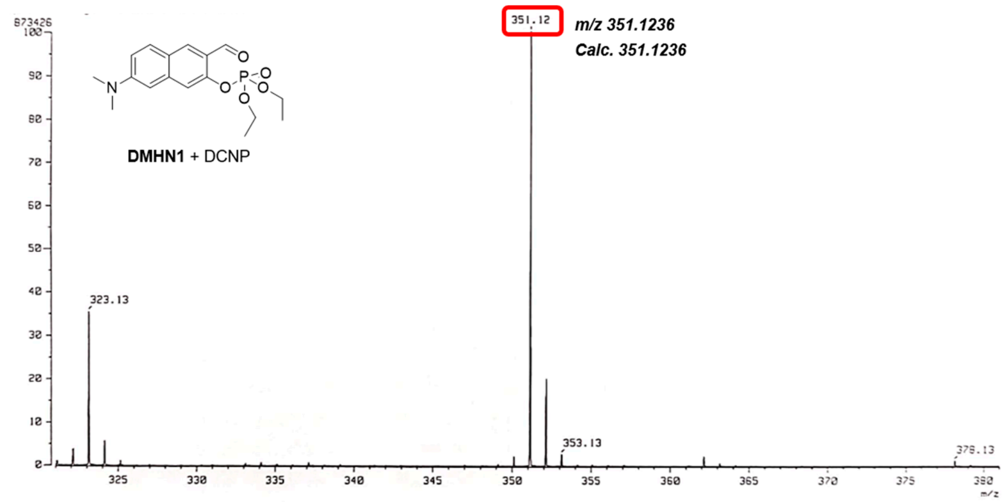

© 2019 by the authors. Licensee MDPI, Basel, Switzerland. This article is an open access article distributed under the terms and conditions of the Creative Commons Attribution (CC BY) license (http://creativecommons.org/licenses/by/4.0/).
